# 2-[(5-Amino-3-methyl-1-phenyl-1*H*-pyrazol-4-yl)(4-chloro­phen­yl)meth­yl]malononitrile

**DOI:** 10.1107/S1600536808011562

**Published:** 2008-04-26

**Authors:** Xin-Ying Zhang, Xiao-Yan Li, Xia Wang, Dong-Fang Li, Xue-Sen Fan

**Affiliations:** a School of Chemical and Environmental Sciences, Henan Key Laboratory for Environmental Pollution Control, Henan Normal University, Xinxiang, Henan 453007, People’s Republic of China

## Abstract

In the crystal structure of the title compound, C_20_H_16_ClN_5_, the dihedral angle between the pyrazole ring and the phenyl ring is 54.7 (1)° and that between the pyrazole ring and the chloro-substituted phenyl ring is 72.4 (1)°. The methyl H atoms are disordered over two positions with site occupancy factors of *ca* 0.7 and 0.3. One amino H is disordered equally over two positions. In the crystal structure, the mol­ecules are linked *via* inter­molecular N—H⋯N hydrogen bonding.
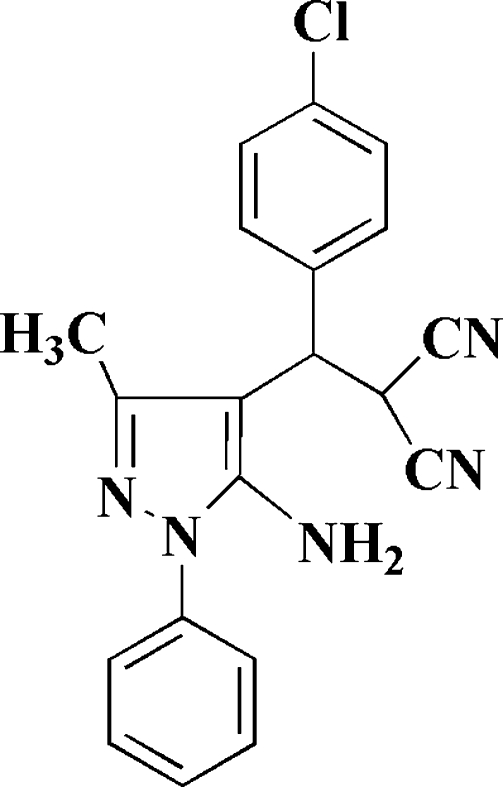

## Experimental

### 

#### Crystal data

C_20_H_16_ClN_5_
*M* *_r_* = 361.83
Orthorhombic, 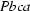

*a* = 10.4700 (11) Å
*b* = 14.0482 (15) Å
*c* = 25.409 (3) Å
*V* = 3737.3 (7) Å^3^
*Z* = 8
Mo *K*α radiation
μ = 0.22 mm^−1^
*T* = 294 (2) K
0.49 × 0.48 × 0.45 mm

#### Data collection

Bruker SMART CCD area-detector diffractometer
Absorption correction: multi-scan (*SADABS*; Bruker, 1997 ▶) *T*_min_ = 0.901, *T*_max_ = 0.908
32456 measured reflections
4661 independent reflections
3055 reflections with *I* > 2σ(*I*)
*R*_int_ = 0.033

#### Refinement

*R*[*F* ^2^ > 2σ(*F* ^2^)] = 0.041
*wR*(*F* ^2^) = 0.118
*S* = 1.03
4661 reflections
237 parameters
H-atom parameters constrained
Δρ_max_ = 0.18 e Å^−3^
Δρ_min_ = −0.32 e Å^−3^

### 

Data collection: *SMART* (Bruker, 1997 ▶); cell refinement: *SAINT* (Bruker, 1997 ▶); data reduction: *SAINT*; program(s) used to solve structure: *SHELXS97* (Sheldrick, 2008 ▶); program(s) used to refine structure: *SHELXL97* (Sheldrick, 2008 ▶); molecular graphics: *SHELXTL* (Sheldrick, 2008 ▶); software used to prepare material for publication: *SHELXTL*.

## Supplementary Material

Crystal structure: contains datablocks I, global. DOI: 10.1107/S1600536808011562/nc2101sup1.cif

Structure factors: contains datablocks I. DOI: 10.1107/S1600536808011562/nc2101Isup2.hkl

Additional supplementary materials:  crystallographic information; 3D view; checkCIF report

## Figures and Tables

**Table 1:** Hydrogen-bond geometry (Å, °)

*D*—H⋯*A*	*D*—H	H⋯*A*	*D*⋯*A*	*D*—H⋯*A*
N3—H1*N*3⋯N4^i^	0.86	2.41	3.159 (2)	146
N3—H2*N*3⋯N2^ii^	0.86	2.53	3.325 (2)	155

Symmetry codes: (i) 
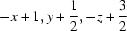
; (ii) 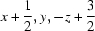.

**Fig. 1.**
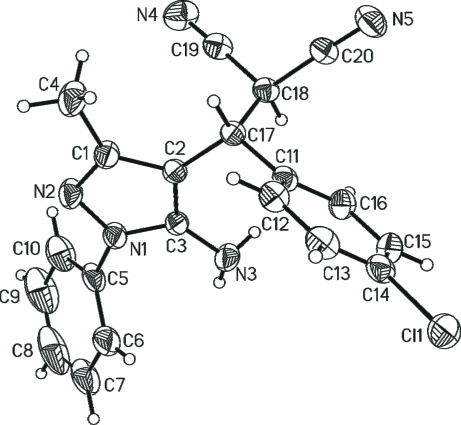
Molecular structure of the title compound, with labelling displacement ellipsoids drawn at the 30% probability level. The disordering of the H atoms is not shown for clarity.

**Fig. 2.**
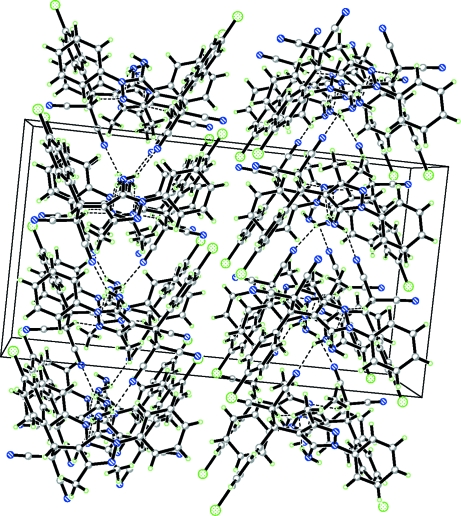
Crystal structure of the title compound with view along the a-axis. Intermolecular N—H···N hydrogen bonding is shown as dashed lines and the disordering of the H atoms is not shown for clarity.

## supplementary crystallographic information

### Comment

The structure determination was undertaken as a part of a project on the synthesis of new pyrazole derivatives. In the title compound the dihedral angle between the pyrazole ring and the non-substituted phenyl ring which is directly connected to the pyrazole ring is 54.7 (1)° and that between the pyrazole ring and the chloro-substituted phenyl ring is 72.4 (1)°. The dihedral angle between the non-substituted and the chloro-substituted phenyl ring amount to 69.7 (1)° (Fig. 1).

In the crystal structure the molecules are connected via intermolecular N—H···N hydrogen bonding between the amino group at N3 and the N atoms N2 and N4 (Fig. 2 and Table 1).

### Experimental

To 1 ml of 1-butyl-3-methylimidazolium tetrafluoroborate ([bmim][BF_4_]), 4-chloroaldehyde (1 mmol), malononitrile (1 mmol) and 5-amino-3-methyl-1-phenylpyrazole (1 mmol) were added. The reaction mixture was stirred at room temperature for 4 h and afterwards extracted five times with 2 ml of diethylether. The ether extracts were combined and concentrated. The obtained residue was recrystallized with 95% ethanol to give the product in a yield of 95% as white solid. Single crystals of the title compound were obtained by slow evaporation of the solvent from a petroleum ether-ethyl ether (1:1 *v*/*v*) solution.

### Refinement

All H atoms were placed in geometrically idealized positions (methyl H atoms are disordered in two orientations) and constrained to ride on their parent atoms, with C—H distances of 0.93 - 0.98 Å and with *U*_iso_(H) = 1.2*U*_eq_(C) (1.5 for methyl H atoms). The N-H H atoms were located in difference map, their bond lengths were set to ideal values and afterwards they were refined using a riding model with *U*_iso_(H) = 1.2*U*_eq_(C). One of the N-H H atoms is disordered and was refined using a split model.

### Crystal data

**Table:**

C_20_H_16_ClN_5_	*D*_x_ = 1.286 Mg m^−^^3^
*M**_r_* = 361.83	Mo *K*α radiation λ = 0.71073 Å
Orthorhombic, *P**b**c**a*	Cell parameters from 8329 reflections
*a* = 10.4700 (11) Å	θ = 2.5–25.8º
*b* = 14.0482 (15) Å	µ = 0.22 mm^−^^1^
*c* = 25.409 (3) Å	*T* = 294 (2) K
*V* = 3737.3 (7) Å^3^	Block, colourless
*Z* = 8	0.49 × 0.48 × 0.45 mm
*F*_000_ = 1504	

### Data collection

**Table:**

Bruker SMART CCD area-detector diffractometer	4661 independent reflections
Radiation source: fine-focus sealed tube	3055 reflections with *I* > 2σ(*I*)
Monochromator: graphite	*R*_int_ = 0.033
*T* = 294(2) K	θ_max_ = 28.4º
φ and ω scans	θ_min_ = 2.5º
Absorption correction: multi-scan(SADABS; Bruker, 1997)	*h* = −14→13
*T*_min_ = 0.901, *T*_max_ = 0.909	*k* = −18→18
32456 measured reflections	*l* = −33→33

### Refinement

**Table:**

Refinement on *F*^2^	Hydrogen site location: inferred from neighbouring sites
Least-squares matrix: full	H-atom parameters constrained
*R*[*F*^2^ > 2σ(*F*^2^)] = 0.041	*w* = 1/[σ^2^(*F*_o_^2^) + (0.0404*P*)^2^ + 1.3457*P*] where *P* = (*F*_o_^2^ + 2*F*_c_^2^)/3
*wR*(*F*^2^) = 0.118	(Δ/σ)_max_ = 0.001
*S* = 1.03	Δρ_max_ = 0.18 e Å^−^^3^
4661 reflections	Δρ_min_ = −0.32 e Å^−^^3^
237 parameters	Extinction correction: SHELXL97 (Sheldrick, 2008), Fc^*^=kFc[1+0.001xFc^2^λ^3^/sin(2θ)]^-1/4^
Primary atom site location: structure-invariant direct methods	Extinction coefficient: 0.0012 (3)
Secondary atom site location: difference Fourier map	

### Special details

**Table:**

Geometry. All e.s.d.'s (except the e.s.d. in the dihedral angle between two l.s. planes)are estimated using the full covariance matrix. The cell e.s.d.'s are takeninto account individually in the estimation of e.s.d.'s in distances, anglesand torsion angles; correlations between e.s.d.'s in cell parameters are onlyused when they are defined by crystal symmetry. An approximate (isotropic)treatment of cell e.s.d.'s is used for estimating e.s.d.'s involving l.s. planes.
Refinement. Refinement of *F*^2^ against ALL reflections. The weighted *R*-factor *wR* andgoodness of fit *S* are based on *F*^2^, conventional *R*-factors *R* are basedon *F*, with *F* set to zero for negative *F*^2^. The threshold expression of*F*^2^ > σ(*F*^2^) is used only for calculating *R*-factors(gt) *etc*. and isnot relevant to the choice of reflections for refinement. *R*-factors basedon *F*^2^ are statistically about twice as large as those based on *F*, and *R*-factors based on ALL data will be even larger.

### Fractional atomic coordinates and isotropic or equivalent isotropic displacement parameters (Å^2^)

**Table:**

	*x*	*y*	*z*	*U*_iso_*/*U*_eq_	Occ. (<1)
Cl1	0.34596 (7)	1.04230 (4)	0.53076 (2)	0.0837 (2)	
N1	0.27144 (14)	0.71251 (10)	0.78626 (5)	0.0509 (4)	
N2	0.17726 (15)	0.64522 (12)	0.77803 (6)	0.0605 (4)	
N3	0.43568 (16)	0.79533 (11)	0.74011 (5)	0.0623 (4)	
N4	0.4155 (2)	0.44105 (12)	0.68772 (7)	0.0751 (5)	
N5	0.52582 (19)	0.58634 (13)	0.54493 (7)	0.0748 (5)	
C1	0.18531 (16)	0.62494 (13)	0.72710 (6)	0.0512 (4)	
C2	0.28333 (15)	0.67629 (11)	0.70228 (5)	0.0414 (3)	
C3	0.33723 (15)	0.73171 (11)	0.74145 (6)	0.0429 (4)	
C4	0.0940 (2)	0.55545 (17)	0.70288 (8)	0.0772 (7)	
H4A	0.0613	0.5811	0.6706	0.116*	0.73
H4B	0.0245	0.5440	0.7267	0.116*	0.73
H4C	0.1376	0.4967	0.6958	0.116*	0.73
H4D	0.0876	0.5001	0.7249	0.116*	0.27
H4E	0.1244	0.5372	0.6687	0.116*	0.27
H4F	0.0114	0.5845	0.6996	0.116*	0.27
C5	0.29537 (17)	0.74496 (15)	0.83845 (6)	0.0579 (5)	
C6	0.2963 (2)	0.84064 (18)	0.84956 (9)	0.0847 (7)	
H6	0.2812	0.8854	0.8233	0.102*	
C7	0.3206 (3)	0.8691 (3)	0.90156 (14)	0.1220 (14)	
H7	0.3232	0.9335	0.9099	0.146*	
C8	0.3407 (3)	0.8023 (4)	0.94005 (12)	0.1401 (18)	
H8	0.3567	0.8218	0.9744	0.168*	
C9	0.3375 (3)	0.7080 (3)	0.92837 (10)	0.1239 (13)	
H9	0.3504	0.6632	0.9548	0.149*	
C10	0.3151 (2)	0.6782 (2)	0.87746 (8)	0.0846 (7)	
H10	0.3133	0.6136	0.8695	0.102*	
C11	0.33508 (15)	0.76013 (11)	0.61584 (5)	0.0415 (3)	
C12	0.22572 (17)	0.79784 (13)	0.59298 (6)	0.0516 (4)	
H12	0.1491	0.7647	0.5956	0.062*	
C13	0.2283 (2)	0.88387 (14)	0.56630 (6)	0.0588 (5)	
H13	0.1545	0.9082	0.5510	0.071*	
C14	0.3417 (2)	0.93244 (12)	0.56298 (6)	0.0550 (5)	
C15	0.45228 (19)	0.89664 (13)	0.58425 (7)	0.0569 (5)	
H15	0.5287	0.9299	0.5811	0.068*	
C16	0.44836 (17)	0.81010 (12)	0.61058 (6)	0.0510 (4)	
H16	0.5230	0.7853	0.6249	0.061*	
C17	0.32395 (14)	0.66623 (11)	0.64546 (5)	0.0401 (3)	
H17	0.2568	0.6296	0.6278	0.048*	
C18	0.44800 (16)	0.60557 (11)	0.64192 (6)	0.0444 (4)	
H18	0.5153	0.6391	0.6613	0.053*	
C19	0.42937 (18)	0.51193 (13)	0.66700 (6)	0.0525 (4)	
C20	0.49158 (17)	0.59315 (12)	0.58707 (7)	0.0511 (4)	
H1N3	0.4639	0.8173	0.7695	0.061*	
H2N3	0.5027	0.7693	0.7270	0.061*	0.50
H3N3	0.4647	0.8134	0.7101	0.061*	0.50

### Atomic displacement parameters (Å^2^)

**Table:**

	*U*^11^	*U*^22^	*U*^33^	*U*^12^	*U*^13^	*U*^23^
Cl1	0.1235 (5)	0.0600 (3)	0.0677 (3)	0.0238 (3)	0.0273 (3)	0.0234 (2)
N1	0.0586 (9)	0.0588 (8)	0.0354 (7)	−0.0138 (7)	0.0064 (6)	−0.0075 (6)
N2	0.0636 (9)	0.0760 (11)	0.0418 (8)	−0.0252 (8)	0.0098 (7)	−0.0067 (7)
N3	0.0761 (11)	0.0659 (9)	0.0448 (8)	−0.0308 (8)	0.0096 (7)	−0.0120 (7)
N4	0.1013 (14)	0.0569 (10)	0.0669 (11)	0.0076 (9)	−0.0131 (10)	0.0121 (8)
N5	0.0918 (13)	0.0807 (12)	0.0518 (9)	0.0059 (10)	0.0158 (9)	−0.0074 (8)
C1	0.0543 (10)	0.0587 (10)	0.0405 (8)	−0.0137 (8)	0.0015 (7)	−0.0033 (7)
C2	0.0474 (8)	0.0430 (8)	0.0338 (7)	−0.0020 (7)	0.0003 (6)	−0.0009 (6)
C3	0.0505 (9)	0.0416 (8)	0.0365 (7)	−0.0045 (7)	0.0040 (6)	−0.0021 (6)
C4	0.0765 (14)	0.0976 (16)	0.0575 (11)	−0.0416 (12)	0.0044 (10)	−0.0101 (11)
C5	0.0542 (10)	0.0810 (13)	0.0384 (8)	−0.0124 (9)	0.0110 (7)	−0.0155 (8)
C6	0.0862 (16)	0.0897 (16)	0.0782 (14)	−0.0306 (13)	0.0300 (12)	−0.0365 (12)
C7	0.102 (2)	0.157 (3)	0.107 (2)	−0.063 (2)	0.0500 (18)	−0.088 (2)
C8	0.091 (2)	0.268 (5)	0.0613 (17)	−0.046 (3)	0.0176 (15)	−0.071 (3)
C9	0.112 (2)	0.220 (4)	0.0398 (12)	0.002 (3)	0.0031 (13)	−0.0061 (18)
C10	0.0911 (17)	0.120 (2)	0.0427 (10)	0.0025 (15)	0.0073 (10)	−0.0001 (12)
C11	0.0507 (9)	0.0458 (8)	0.0281 (7)	0.0033 (7)	0.0007 (6)	−0.0010 (6)
C12	0.0513 (10)	0.0586 (10)	0.0449 (9)	0.0051 (8)	−0.0033 (7)	0.0022 (7)
C13	0.0670 (12)	0.0632 (11)	0.0461 (9)	0.0200 (10)	−0.0025 (8)	0.0052 (8)
C14	0.0803 (13)	0.0494 (9)	0.0353 (8)	0.0139 (9)	0.0115 (8)	0.0056 (7)
C15	0.0649 (11)	0.0555 (10)	0.0503 (10)	−0.0040 (9)	0.0091 (8)	0.0076 (8)
C16	0.0514 (10)	0.0555 (10)	0.0462 (9)	0.0005 (8)	−0.0012 (7)	0.0083 (7)
C17	0.0450 (8)	0.0438 (8)	0.0315 (7)	−0.0024 (7)	−0.0039 (6)	−0.0020 (6)
C18	0.0516 (9)	0.0471 (9)	0.0345 (7)	0.0018 (7)	−0.0056 (6)	−0.0022 (6)
C19	0.0623 (11)	0.0530 (10)	0.0421 (8)	0.0088 (8)	−0.0079 (8)	−0.0005 (7)
C20	0.0560 (10)	0.0515 (10)	0.0457 (9)	0.0062 (8)	0.0001 (8)	−0.0027 (7)

### Geometric parameters (Å, °)

**Table:**

Cl1—C14	1.7477 (17)	C6—H6	0.9300
N1—C3	1.3577 (19)	C7—C8	1.371 (5)
N1—N2	1.3819 (19)	C7—H7	0.9300
N1—C5	1.424 (2)	C8—C9	1.359 (5)
N2—C1	1.328 (2)	C8—H8	0.9300
N3—C3	1.365 (2)	C9—C10	1.379 (3)
N3—H1N3	0.8602	C9—H9	0.9300
N3—H2N3	0.8587	C10—H10	0.9300
N3—H3N3	0.8595	C11—C16	1.385 (2)
N4—C19	1.136 (2)	C11—C12	1.389 (2)
N5—C20	1.133 (2)	C11—C17	1.523 (2)
C1—C2	1.404 (2)	C12—C13	1.386 (2)
C1—C4	1.499 (2)	C12—H12	0.9300
C2—C3	1.384 (2)	C13—C14	1.372 (3)
C2—C17	1.5117 (19)	C13—H13	0.9300
C4—H4A	0.9600	C14—C15	1.373 (3)
C4—H4B	0.9600	C15—C16	1.388 (2)
C4—H4C	0.9600	C15—H15	0.9300
C4—H4D	0.9600	C16—H16	0.9300
C4—H4E	0.9600	C17—C18	1.556 (2)
C4—H4F	0.9600	C17—H17	0.9800
C5—C6	1.373 (3)	C18—C19	1.475 (2)
C5—C10	1.380 (3)	C18—C20	1.477 (2)
C6—C7	1.404 (4)	C18—H18	0.9800
			
C3—N1—N2	111.78 (12)	C7—C6—H6	120.9
C3—N1—C5	128.89 (14)	C8—C7—C6	120.3 (3)
N2—N1—C5	119.03 (13)	C8—C7—H7	119.8
C1—N2—N1	104.41 (13)	C6—C7—H7	119.8
C3—N3—H1N3	118.2	C9—C8—C7	120.5 (3)
C3—N3—H2N3	110.3	C9—C8—H8	119.8
H1N3—N3—H2N3	102.1	C7—C8—H8	119.8
C3—N3—H3N3	118.9	C8—C9—C10	120.3 (3)
H1N3—N3—H3N3	122.9	C8—C9—H9	119.8
H2N3—N3—H3N3	59.4	C10—C9—H9	119.8
N2—C1—C2	111.96 (14)	C9—C10—C5	119.6 (3)
N2—C1—C4	119.96 (16)	C9—C10—H10	120.2
C2—C1—C4	128.07 (15)	C5—C10—H10	120.2
C3—C2—C1	105.31 (13)	C16—C11—C12	118.19 (15)
C3—C2—C17	128.66 (14)	C16—C11—C17	123.53 (14)
C1—C2—C17	125.92 (14)	C12—C11—C17	118.29 (14)
N1—C3—N3	122.28 (14)	C13—C12—C11	121.41 (17)
N1—C3—C2	106.52 (13)	C13—C12—H12	119.3
N3—C3—C2	131.19 (14)	C11—C12—H12	119.3
C1—C4—H4A	109.5	C14—C13—C12	118.72 (17)
C1—C4—H4B	109.5	C14—C13—H13	120.6
H4A—C4—H4B	109.5	C12—C13—H13	120.6
C1—C4—H4C	109.5	C13—C14—C15	121.56 (16)
H4A—C4—H4C	109.5	C13—C14—Cl1	119.35 (15)
H4B—C4—H4C	109.5	C15—C14—Cl1	119.09 (16)
C1—C4—H4D	109.5	C14—C15—C16	119.04 (18)
H4A—C4—H4D	141.1	C14—C15—H15	120.5
H4B—C4—H4D	56.3	C16—C15—H15	120.5
H4C—C4—H4D	56.3	C11—C16—C15	121.05 (16)
C1—C4—H4E	109.5	C11—C16—H16	119.5
H4A—C4—H4E	56.3	C15—C16—H16	119.5
H4B—C4—H4E	141.1	C2—C17—C11	114.36 (12)
H4C—C4—H4E	56.3	C2—C17—C18	109.95 (12)
H4D—C4—H4E	109.5	C11—C17—C18	112.45 (12)
C1—C4—H4F	109.5	C2—C17—H17	106.5
H4A—C4—H4F	56.3	C11—C17—H17	106.5
H4B—C4—H4F	56.3	C18—C17—H17	106.5
H4C—C4—H4F	141.1	C19—C18—C20	110.08 (14)
H4D—C4—H4F	109.5	C19—C18—C17	110.68 (14)
H4E—C4—H4F	109.5	C20—C18—C17	112.15 (13)
C6—C5—C10	121.1 (2)	C19—C18—H18	107.9
C6—C5—N1	120.4 (2)	C20—C18—H18	107.9
C10—C5—N1	118.53 (19)	C17—C18—H18	107.9
C5—C6—C7	118.2 (3)	N4—C19—C18	177.99 (18)
C5—C6—H6	120.9	N5—C20—C18	178.0 (2)

### Hydrogen-bond geometry (Å, °)

**Table:**

*D*—H···*A*	*D*—H	H···*A*	*D*···*A*	*D*—H···*A*
N3—H1N3···N4^i^	0.86	2.41	3.159 (2)	146
N3—H2N3···N2^ii^	0.86	2.53	3.325 (2)	155

Symmetry codes: (i) −*x*+1, *y*+1/2, −*z*+3/2; (ii) *x*+1/2, *y*, −*z*+3/2.
